# From Acute Phase to Long COVID: A Cross-Sectional Study of the Epidemiological Profile and Clinical Evaluation of SARS-CoV-2 Infection in Employees at a Pediatric Hospital

**DOI:** 10.3390/jcm12134325

**Published:** 2023-06-27

**Authors:** Marielen Ribeiro Tavares da Silva, Ana Paula Costa, Amanda Almeida da Luz, Caio Henrique Pelaio, Felipe Baleche Cruz, Giovanna Fischer Steil, Heloisa Ihle Garcia Giamberardino, Carolina Prando

**Affiliations:** 1 Faculdades Pequeno Príncipe, Curitiba 80230-020, PR, Brazil; marielen.silva@aluno.fpp.edu.br (M.R.T.d.S.);; 2 Instituto de Pesquisa Pelé Príncipe, Curitiba 80250-060, PR, Brazil; 3 Hospital Pequeno Príncipe, Curitiba 80250-060, PR, Brazil; ana.costa@hpp.org.br (A.P.C.); heloisa.ihle@hpp.org.br (H.I.G.G.); 4 Biomedical and Clinical Analyses Department, Centro Universitário Curitiba, Curitiba 80220-181, PR, Brazil; 5 Medical School, Faculdade Evangélica Mackenzie do Paraná, Curitiba 80730-000, PR, Brazil

**Keywords:** COVID-19, long COVID, healthcare professionals, reinfection, occupational medicine

## Abstract

Background: The coronavirus disease 2019 (COVID-19) pandemic began in Brazil on 26 February 2020. By 6 May 2023, 37.4 million cases had been confirmed, causing 701 thousand deaths in the country. We aimed to describe the epidemiological profile and clinical development of COVID-19 cases among the employees of a health institution, from acute infection to long COVID. Methods: This was a longitudinal study using a retrospective and prospective approach via questionnaires referring to epidemiological investigation, which was the inclusion criteria, and about long-term symptoms. Results: A total of 809 employees were detected with SARS-CoV-2 infection via RT-PCR, 466 of them answered the epidemiological investigation, and 101 completed the Long COVID Symptom Questionnaire. The most commonly affected employees were women (88.6%) working in patient care (68.6%). Headache, myalgia, cough, odynophagia, and runny nose were the most frequent symptoms. Only three employees (0.6%) required hospitalization, while the other employees required outpatient management due to mild symptoms. We identified 19 (4.1%) cases of reinfection, and 42 (41.6%) employees reported long-term symptoms, such as myalgia, dyspnea, and headache. Conclusions: Although most cases were mild with good outcomes, long COVID cases identified are noteworthy, as these symptoms may impact quality of life even months after SARS-CoV-2 infection.

## 1. Introduction

The coronavirus disease 2019 (COVID-19) pandemic, caused by the new coronavirus, severe acute respiratory syndrome coronavirus (SARS-CoV)-2, began in Brazil on 26 February 2020. By 6 May 2023, 37.4 million cases had been confirmed in the country, causing 701,000 deaths. Among the confirmed cases, 2.9 million occurred in Paraná, with 46,000 confirmed deaths [1].

Many routine adaptations are necessary to prevent the spread of the infection. These include mandatory isolation, social distancing, the use of masks, hand hygiene, and other protective measures. Concurrently, health systems must operate at their maximum capacity [2] as health professionals face potentially fatal and unknown diseases.

A systematic review showed that although healthcare professionals recognized being exposed to the risk of contracting the virus, they accepted this inherent fact to be part of their professions [3]. Therefore, the safety of these professionals in their work environments must be guaranteed, as they are essential when facing extreme situations such as pandemics [2].

In this context, SARS-CoV-2 reinfections and the manifestation of long-term symptoms, known as long COVID, have also gained notoriety in recent studies [4,5,6,7,8], as they affect the quality of life and health even months after the first acute episode of infection.

Therefore, health services must monitor the epidemiology of COVID-19 and understand the factors involved in the occupational risk of their employees in the face of the disease to avoid and reduce transmission. It is also necessary to ensure they receive the necessary assistance and support if they test positive for COVID-19. Therefore, we aimed to describe the epidemiological profile and clinical development of COVID-19 cases among the employees of a health institution, from acute infection to long COVID.

## 2. Materials and Methods

This was a longitudinal study using a retrospective and prospective approach, performed from March 2020 to December 2021, and was approved by the Ethics Committee (No. 49886221.6.0000.0097). This study was conducted in a reference teaching hospital for pediatric COVID-19 patients in Curitiba, southern Brazil. The hospital had 372 beds, with 62 beds designated for intensive care units. During the study period, there were 2275 employees in the institution. We considered all employees, including frontline healthcare professionals in the fight against COVID-19 and professionals in administrative functions, as participants in this study.

To combat COVID-19, the hospital’s occupational medicine service implemented a protocol for tracking positive cases and asymptomatic COVID-19 contacts through an outpatient clinic exclusively dedicated to caring for employees. This service aimed to isolate and monitor the development of these cases and prevent the spread of the disease to co-workers and patients.

By default, the screening involved reverse transcription-polymerase chain reaction (RT-PCR) examination for diagnostic confirmation of COVID-19. Once the case was confirmed, the employee was immediately requested to complete a questionnaire referring to an epidemiological investigation. The inclusion criterion for the retrospective analysis of the acute phase of infection was a questionnaire response. After an interval of 6 months to 1 year, we sent an online questionnaire via Google Forms about long-term symptoms, and this response was the inclusion criterion for the prospective analysis. Answering the surveys was voluntary in both cases, with informed consent provided before sending responses. This flow was implemented for new and possible reinfection cases. Cases of reinfection were defined as two positive PCR results within a minimum period of 90 days between the first and second collections.

Statistical analyses were performed using an R statistical computing environment [9]. The main R packages used were {readxl} [10], {dplyr} [11], {tidyr} [12], {ggplot2} [13], {purrr} [14], {haven} [15], and {broom} [16].

We evaluated the differences between frequencies using Fisher’s exact test [17,18] and differences between means using Student’s *t*-test. Statistical significance was set at 95% with *p* < 0.05.

## 3. Results

**Figure 1:**
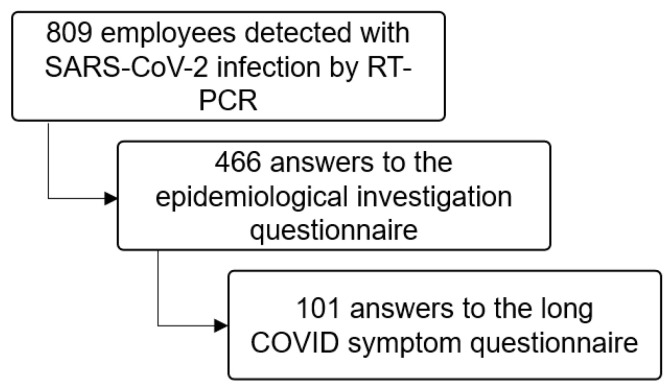
Flowchart of study participants: staff at a pediatric hospital who tested positive for severe acute respiratory syndrome coronavirus-2 reverse transcription-polymerase chain reaction between March 2020 and December 2021.

A total of 809 employees were detected with SARS-CoV-2 infection via RT-PCR, and 466 of them answered the epidemiological investigation questionnaire. Of these, 101 completed the Long COVID Symptom Questionnaire (Figure 1).

### 3.1. Epidemiological Profile

Women accounted for 88.6% of the positive cases (*p* < 0.01), and the most affected age group was 30–39 years (32.4%), followed by the 40–49 (27.0%), 20–29 (23.2%), 50–59 (15.9%), and >60 years age groups (1.5%) (*p* < 0.01). Responses to the epidemiological investigation questionnaire are presented in Table 1. Although more than half of the employees who tested positive for COVID-19 worked in patient care (68.6%), only 21.5% were directly involved in caring for patients with COVID-19, and 20.8% reported incidents of direct exposure to aerosols or biological agents. There was a significant difference in infections among those directly involved in caring for patients with COVID-19 (*p* < 0.01). Half of them claimed contact with persons living in the same house who were positive for COVID-19, whereas 27.7% reported having a confirmed positive case in the same working area. Most employees reported following guidelines for preventive measures against COVID-19, such as social distancing (98.9%), not participating in social events (94.9%), and not receiving visitors to their homes (61.8%). However, most could not avoid attending essential service environments (81.5%).

The institution implemented compulsory personal protective equipment (PPE) training, which ensured 94.6% proper and regular use of such equipment and 81.25% training participation. The frequencies of the most critical items reported are listed in Table 2.

### 3.2. Symptoms

Most employees (95.5%) reported symptoms associated with infection. Headache, myalgia, cough, odynophagia, and runny nose were the most frequent symptoms (Table 3). Additionally, 21 of them (4.5%) reported no symptoms, being classified as asymptomatic.

Approximately 10% of the employees reported having comorbidities. Systemic arterial hypertension (6.2%) was the most common comorbidity, followed by asthma (2.6%), hypothyroidism (2.0%), diabetes (1.5%), and obesity (0.4%). Only three employees (0.6%) required hospitalization to monitor symptoms. The other ones reported mild symptoms requiring outpatient management.

### 3.3. Reinfection

Nineteen cases of reinfection (4.1%) were identified. Headache, coryza, odynophagia, cough, and myalgia were among the most frequent symptoms, with no significant differences between the clinical manifestations of the first and second infections (Table 4). None of the participants reported hospitalization to control their clinical manifestations. The mean interval between the first and second infections was 269.8 days (range, 111–460 days).

### 3.4. Long COVID

Of the 101 employees who responded to the online questionnaire, 42 (41.6%) reported long-term symptoms (Table 5), such as myalgia (15.8%), dyspnea (13.9%), and headache (13.9%). When the most frequent symptoms of the acute phase and long COVID were compared (Table 6), tiredness manifested only in the acute phase of the disease (*p* < 0.01). Headache and odynophagia were more frequent symptoms in the acute phase (*p* < 0.01). Interestingly, dyspnea was more frequent in the long COVID (*p* < 0.01). There was no difference in myalgia, anosmia, and ageusia frequency among acute and long COVID phases.

## 4. Discussion

In this cohort study, we analyzed the epidemiological profile of SARS-CoV-2 infection in 466 employees in a Brazilian pediatric hospital through clinical data on acute infection and long COVID questionnaires. We observed that most cases occurred among employees who worked in the care area, especially in the nursing team, developing entirely with a mild form of the disease, with 19 cases of reinfection. Long-term symptom data from 101 participants showed that 57.4% had not fully recovered within 6–12 months of infection. To the best of our knowledge, this is the first study to address long COVID in pediatric hospital healthcare professionals.

In this study, most people infected (88.6%) were women. However, this can be explained by the fact that women were the majority among the institution’s employees (85%). Women also represent the majority of cases in other institutions [19]. The most affected age group was 30 to 39 years, similar to a systematic review that included 119,883 employees and observed that 38 years old was the mean participants’ age [20].

Nurses accounted for nearly half of the COVID-19-positive participants in the present study. In a systematic review of 152,888 positive cases, this professional class accounted for 38.6% of cases, representing the highest proportion among professionals [19]. In another study, being a nursing technician was classified as a risk factor for COVID-19 among health professionals, indicating that they were more vulnerable to infection by SARS-CoV-2 [21]. This can be explained by the fact that nursing professionals in the present study represent 38.6% of the institution’s staff. In addition, they spent most of their time providing direct assistance and contacting patients.

The use of PPE has been widely discussed regarding the availability and training institutions offer. Unlike other health institutions, where up to 51.4% of professionals reported not having received training in PPE and 47.7% reported a shortage of PPE, in this study, 81.2% reported having received adequate training and 94.6% reported having used it habitually and correctly [21,22]. Despite this, the employees tested positive for COVID-19. It is essential to emphasize that the employee responses were subjective, in addition to having other potential sources of infection that are relevant, such as frequenting markets, pharmacies, and shops (81.5%), and using public transport to travel to work (56.9%). In addition, 51.1% reported having infected persons in the same home environment, indicating that the disease was already at a high rate of community transmission.

Only 12.5% of employees had comorbidities, and no correlation was found between the presence of comorbidities and the need for hospitalization, which was extremely low (0.6%) in this study. Other studies in health institutions reported that up to 34% of employees with comorbidities were affected by COVID-19, with a higher hospitalization rate between 11.3% and 12.8% [7,20,21,23]. It is important to emphasize that the local study was conducted in the southern region of Brazil, which has health indicators such as “Hospitalization for Primary Care-sensitive conditions” that are better than the Brazilian national average.

The eight most frequent symptoms in this study were headache, myalgia, cough, odynophagia, runny nose, fever, anosmia, and ageusia. In a systematic review that included more than 119,000 healthcare professionals, the most reported symptoms were fever, cough, fatigue, sputum, headache, odynophagia, diarrhea, nausea, and vomiting [20]. Headache, cough, odynophagia, and fever were the most common symptoms in the two studies. In terms of outcomes, the cases in the present study were milder, with less than 1% hospitalizations and no deaths, compared to the same study, which had 15.1% hospitalizations and 1.5% deaths [20].

We observed a reinfection rate of 4.1% with no statistically significant difference between the symptoms reported for the two infections, with headache being the predominant symptom in both studies. Previous studies have reported reinfection rates ranging from 1.9% and 7.9% among professionals collaborating with healthcare services [24,25,26,27]. One of these studies, a case–control study that reported 33 cases of reinfection, also presented headache as the primary symptom in both infections, but with an infectious condition and more severe outcomes requiring mechanical ventilation and death during the second infection. This difference may have occurred because of comorbidities, as 45.5% of the reported cases had some comorbidities [27].

The condition of long-term symptoms post-COVID-19 infection, known as long COVID, has generated increasing interest and discussion among researchers [4,5,6,7,8] because it affects the quality of life and health of affected individuals. The causes for the maintenance or presentation of new symptoms, even months after acute infection by SARS-CoV-2, are still not fully elucidated, with the hypotheses that they may occur because of viral persistence in the body, autoimmune mechanisms, and dysregulation of the immune system mediated by SARS-CoV-2 superantigens [28].

In some studies conducted with health professionals, the frequency of reporting long-term symptoms ranged from 24.2% to 45% [6,7]. In the present study, this rate was similar, at 41.6%, among the participants who answered the questionnaire, with myalgia (15.8%), dyspnea (13.9%), headache (13.9%), and anosmia (7.9%) being the most frequent symptoms. We identified dyspnea as relevant in long COVID even though the participant has not presented this symptom in the acute phase. Other studies have reported relevant neurological symptoms such as anxiety, insomnia, and difficulty concentrating [6,29,30]. In the present study, participants reported neurological symptoms, such as mental confusion (6.9%), forgetfulness (3.9%), and difficulty concentrating (0.9%), but with a lower prevalence.

Since the study had a retrospective approach in the acute phase of infection, collecting epidemiological data was limited. Some data were captured in a Microsoft Excel spreadsheet filled out directly by an employee. Thus, information from critically ill patients who were hospitalized with no means of accessing and answering the questionnaire may have been lost. In addition, vaccination started in the institution in February 2021, and it was not considered in our analyses.

This study assessed the acute, long COVID infection of a large group of employees at a pediatric health institution, which could provide a better understanding of the associated factors.

## 5. Conclusions

Although most cases presented in this study were mild with good outcomes, reports of the long-term symptoms identified are noteworthy. Understanding this fact is vital for mapping actions supporting employees, as these symptoms may impact their quality of life even months after SARS-CoV-2 infection.

Thus, even in a mild pandemic scenario, it is necessary to consider measures to protect, support, and assist employees through occupational health management. Future studies considering long COVID alone need to be developed to detail these conditions in this population.

## Figures and Tables

**Table 1:** Epidemiological profile of employees at a pediatric hospital in southern Brazil who tested positive for severe acute respiratory syndrome coronavirus-2 between March 2020 and December 2021.

Answers to the Epidemiological Investigation Questionnaire	Yes	No	Not Answered
Work in patient care activities	319 (68.5%)	146 (31.3%)	1 (0.2%)
Direct assistance to persons suspected or confirmed to have COVID-19	100 (21.5%)	365 (78.3%)	1 (0.2%)
Report of incidents with direct exposure to aerosols/biological agents within the institution?	97 (20.8%)	364 (78.1%)	5 (1.1%)
Confirmation of positive cases that occurred in the sector you worked in the last 15 days	129 (27.7%)	336 (72.1%)	1 (0.2%)
Activity of working in a second health institution	108 (23.2%)	357 (76.6%)	1 (0.2%)
Confirmation of positive cohabitant for COVID-19 during the last 15 days	238 (51.1%)	228 (48.9%)	0 (0.0%)
Receipt of visits at home in the last 15 days	177 (38.0%)	288 (61.8%)	1 (0.2%)
Attended markets, pharmacies, and shops	380 (81.5%)	86 18.5%)	0 (0.0%)
Participation in a social event	23 (4.9%)	443 (95.1%)	0 (0.0%)
Followed social distancing, mask-wearing, and hand hygiene	461 (98.9%)	5 (1.1%)	0 (0.0%)
Use of public transport as a means of commuting to work	265 (56.9%)	201 (43.1%)	0 (0.0%)

Abbreviations: COVID-19, coronavirus disease 2019.

**Table 2:** Personal protective equipment used by employees of a pediatric hospital in southern Brazil during the coronavirus disease 2019 pandemic from March 2020 to December 2021.

PPE	Frequency of Use
N95 Mask	275 (59.0%)
Face shield	181 (38.8%)
Disposable mask	142 (30.5%)
Glasses	97 (20.8%)
Fabric mask	84 (18%)

Abbreviations: PPE, personal protective equipment.

**Table 3:** Symptoms mentioned in the acute phase of infection by the severe acute respiratory syndrome coronavirus-2 in employees at a referral hospital for pediatric coronavirus disease 2019 care.

Symptom	Frequency
Headache	277 (59.4%)
Myalgia	216 (46.4%)
Cough	169 (36.3%)
Odynophagia	168 (36.1%)
Runny nose	158 (33.9%)
Fever	96 (20.6%)
Anosmia	92 (19.7%)
Ageusia	81 (17.4%)
Diarrhea	62 (13.3%)
Fatigue/asthenia/fatigue	61 (13.1%)
Nasal congestion	60 (12.9%)
Nausea/vomiting	54 (11.6%)
Eye symptoms ^(1)^	53 (11.4%)
Sneezing	44 (9.4%)
Dyspnea	44 (9.4%)
Lack of appetite	37 (7.9%)
Back pain	34 (7.3%)
Chill	34 (7.3%)
Chest pain	28 (6.0%)
Abdominal pain	22 (4.7%)
Sweating	18 (3.9%)
Otalgia	12 (2.6%)
General malaise	12 (2.6%)
Others ^(2)^	19 (4.1%)

Note: ^(1)^ pain, irritation, tearing, burning, redness, and itching; ^(2)^ less than 1.0% of cases of tremors, arthralgia, dizziness, bitter taste in the mouth, hypothermia, throat clearance, and tachycardia.

**Table 4:** Frequency of symptoms in the first and second infection by severe acute respiratory syndrome coronavirus-2 in employees of a pediatric hospital in southern Brazil.

Symptom	First Infection	Reinfection
Asymptomatic	4 (21.1%)	2 (10.5%)
Chill	3 (15.8%)	1 (5.2%)
Headache	8 (42.1%)	5 (26.3%)
Nasal congestion	4 (21.1%)	1 (5.2%)
Runny nose	6 (31.6%)	5 (26.3%)
Diarrhea	2 (10.5%)	3 (15.8%)
Dyspnea	1 (5.2%)	2 (10.5%)
Abdominal pain	1 (5.2%)	0 (0.0%)
Odynophagia	6 (3.1%)	3 (15.8%)
Myalgia	6 (3.1%)	3 (15.8%)
Chest pain	0 (0.0%)	2 (10.5%)
Back pain	1 (5.2%)	0 (0.0%)
Sneezing	1 (5.2%)	3 (15.8%)
Fatigue/asthenia	3 (15.8%)	3 (15.8%)
Fever	4 (21.1%)	2 (10.5%)
Malaise	0 (0.0%)	2 (10.5%)
Nausea/vomiting	1 (5.2%)	3 (15.8%)
Anosmia	2 (10.5%)	2 (10.5%)
Ageusia	1 (5.2%)	1 (5.2%)
Ocular symptom	3 (15.8%)	2 (10.5%)
Sweating	1 (5.2%)	0 (0.0%)
Cough	6 (31.6%)	7 (36.8%)

**Table 5:** Long COVID symptoms reported by pediatric referral hospital employees.

Long COVID Symptom	Frequency
Myalgia	16 (15.8%)
Dyspnea	14 (13.9%)
Headache	14 (13.9%)
Anosmia	8 (7.9%)
Ageusia	7 (6.9%)
Mental confusion	7 (6.9%)
Odynophagia	4 (3.9%)
Forgetfulness	4 (3.9%)
Lack of concentration	1 (0.9%)
Diarrhea/abdominal discomfort	3 (2.9%)
Back pain	2 (2.0%)
Cough	1 (1.0%)
Loss of hair	1 (1.0%)
Fatigue	1 (1.0%)
Malaise	1 (1.0%)
Fever	1 (1.0%)

Abbreviations: COVID-19, coronavirus disease 2019.

**Table 6:** Frequency of symptoms present in the acute phase and in long COVID.

Symptoms	Acute Phase (*n* = 101)	Long COVID (*n* = 42)	Fisher’s Exact Test
Tiredness			
Yes	19 (18.8%)	0 (0.0%)	*p* = 0.0010
No	82 (81.2%)	42 (100.0%)	
Headache			
Yes	68 (67.3%)	14 (33.4%)	*p* = 0.0003
No	33 (32.7%)	28 (66.6%)	
Dyspnea			
Yes	6 (5.9%)	14 (33.4%)	*p* < 0.0001
No	95 (94.1%)	28 (66.6%)	
Odynophagia			
Yes	31 (30.7%)	4 (9.6%)	*p* = 0.0095
No	70 (69.3%)	38 (90.4%)	
Myalgia			
Yes	45 (44.6%)	16 (38.3%)	*p* = 0.5783
No	56 (55.4%)	26 (61.7%)	
Anosmia			
Yes	26 (25.8%)	8 (19.1%)	*p* = 0.5184
No	75 (74.2%)	34 (80.9%)	
Ageusia			
Yes	16 (15.9%)	7 (16.7%)	*p* = 1.00
No	85 (84.1%)	35 (83.3%)	

Abbreviations: COVID-19, coronavirus disease 2019.

## Data Availability

The data presented in this study are available on request from the corresponding author.
